# HIV-1 Tat Induces Dysregulation of PGC1-Alpha and Sirtuin 3 Expression in Neurons: The Role of Mitochondrial Biogenesis in HIV-Associated Neurocognitive Disorder (HAND)

**DOI:** 10.3390/ijms242417566

**Published:** 2023-12-17

**Authors:** Izchel Figarola-Centurión, Martha Escoto-Delgadillo, Gracia Viviana González-Enríquez, Juan Ernesto Gutiérrez-Sevilla, Eduardo Vázquez-Valls, Jhonathan Cárdenas-Bedoya, Blanca Miriam Torres-Mendoza

**Affiliations:** 1 Doctorado en Genética Humana, Departamento de Biología Molecular y Genómica, Universidad de Guadalajara, Guadalajara 44340, Mexico; figarola.centurion.izchel@gmail.com; 2 Laboratorio de Inmunodeficiencias y Retrovirus Humanos, Centro de Investigación Biomédica de Occidente, Instituto Mexicano del Seguro Social, Guadalajara 44340, Mexico; juan251995@hotmail.com (J.E.G.-S.); jcbgeneticahumana@hotmail.com (J.C.-B.); 3 Centro Universitario de Ciencias Biológicas y Agropecuarias, Universidad de Guadalajara, Guadalajara 44600, Mexico; 4 Departamento de Disciplinas Filosófico, Metodológicas e Instrumentales, Centro Universitario de Ciencias de la Salud, Universidad de Guadalajara, Guadalajara 44340, Mexico; gvivigoen@gmail.com; 5 Doctorado en Microbiología Médica, Departamento de Microbiología y Patología, Universidad de Guadalajara, Guadalajara 44340, Mexico; 6 Investigación y Calidad de Salud, Secretaría de Salud, Guadalajara 44100, Mexico; eduardovazquezvalls@gmail.com

**Keywords:** HIV, HAND, mitophagy, mitochondrial biogenesis, *Sirt3*, *Pink1*, *Ppargc1a*, *Tert*, mitochondrial dysfunction, telomeric dysfunction

## Abstract

During the antiretroviral era, individuals living with HIV continue to experience milder forms of HIV-associated neurocognitive disorder (HAND). Viral proteins, including Tat, play a pivotal role in the observed alterations within the central nervous system (CNS), with mitochondrial dysfunction emerging as a prominent hallmark. As a result, our objective was to examine the expression of genes associated with mitophagy and mitochondrial biogenesis in the brain exposed to the HIV-1 Tat protein. We achieved this by performing bilateral stereotaxic injections of 100 ng of HIV-1 Tat into the hippocampus of Sprague–Dawley rats, followed by immunoneuromagnetic cell isolation. Subsequently, we assessed the gene expression of *Ppargc1a*, *Pink1*, and *Sirt1-3* in neurons using RT-qPCR. Additionally, to understand the role of *Tert* in telomeric dysfunction, we quantified the activity and expression of *Tert*. Our results revealed that only *Ppargc1a*, *Pink1*, and mitochondrial *Sirt3* were downregulated in response to the presence of HIV-1 Tat in hippocampal neurons. Interestingly, we observed a reduction in the activity of *Tert* in the experimental group, while mRNA levels remained relatively stable. These findings support the compelling evidence of dysregulation in both mitophagy and mitochondrial biogenesis in neurons exposed to HIV-1 Tat, which in turn induces telomeric dysfunction.

## 1. Introduction

HIV-associated neurocognitive disorder (HAND) is an emerging comorbidity that encompasses a spectrum of cognitive impairments, affecting over 40% of individuals living with HIV [1,2]. Even though antiretroviral therapy (ART) lowers the likelihood of developing HAND, the less severe forms persist [1,2] even when the viral load is undetectable [3]. To date, during the ART era, the etiology of HAND appears multifactorial yet remains incompletely elucidated [4,5]. Nevertheless, it has been established that the integrity of the blood–brain barrier is compromised in HAND [6] and that microglia, macrophages [7], and astrocytes [8] can harbor viral genetic material in the central nervous system (CNS) of HIV-controlled infected individuals.

As a result, research efforts have focused on understanding the role of infected glial cells in HAND. The implications in the CNS arise from the fact that these cells fail to provide adequate support to neurons and release proinflammatory cytokines [9,10] and viral proteins that contribute to neurotoxicity [8,11]. Tat, an HIV protein involved in the transactivation of viral transcription [12], is released into the extracellular space [13,14] making it available for uptake by neighboring cells [15]. This viral protein can evade endolysosomal degradation [16,17] and trigger mitochondrial dysfunction in both microglia [18] and neurons [19,20,21].

Mitochondrial dysfunction is a hallmark in neurodegenerative conditions such as Alzheimer’s disease [22] and HAND [23]. Tat-induced alterations in mitochondria include mitochondrial membrane depolarization [19,24], perturbations in calcium homeostasis [19,25], damage to mitochondrial DNA, dysregulation of ATP production [26], and increases mitochondrial reactive oxygen species (ROS) levels [19,25,27]. Under the condition of oxidative stress, telomerase is translocated to the mitochondrial matrix upon phosphorylation [28], and evidence suggests that telomerase improves mitochondrial function by decreasing mitochondrial ROS generation, partly attributed to an elevation of mRNA of antioxidant players such as superoxide dismutase 2 (SOD2) [28]. However, its exact role remains elusive [28,29]. Furthermore, Tat has been shown to decrease its activity in microglia [27]. On the other hand, sirtuins 1–7 (SIRT1-7) are a group of NAD+-dependent deacetylase enzymes actively engaged in a wide range of cellular functions, prominently contributing to the maintenance of redox homeostasis [30]. Specifically, among these sirtuins, SIRT3, which resides in the mitochondria, has a crucial role in ameliorating the impact of ROS [27]. When Tat exposure occurs in microglia, it leads to downregulation of the expression of SIRT3 and antioxidant enzymes [27]. During periods of oxidative stress, cells activate mitophagy as a mechanism to eliminate defective mitochondria [31]. In HIV-1 Tat-mediated mitophagy, the PTEN-induced putative kinase protein 1 (PINK1), a kinase essential to mitophagosome formation, is upregulated in microglia [18]. Nevertheless, defective mitochondria accumulate in microglia [18] and neurons [32].

Alternatively, mitochondrial biogenesis serves as a compensatory mechanism to counterbalance mitochondrial loss. To initiate the formation of new mitochondria, the synthesis of specific nuclear-encoded proteins is necessary. These proteins are under the regulation of peroxisome proliferator-activated receptor-gamma coactivator 1-alpha (PGC1a). PGC1a orchestrates the transcription of crucial proteins like the mitochondrial transcription factor A (TFAM), essential for generating components involved in cellular respiration and the electron transport chain [33,34]. Thus, PGC1a emerges as a central modulator in the process of mitochondrial biogenesis [33,34,35]. Swinton et al. found that PGC1a levels were decreased in postmortem brains of individuals diagnosed with HAND [36], indicating that mitochondrial biogenesis is also altered in this condition. PGC1a is activated by SIRT1 [33], which in turn is inhibited by Tat [30].

SIRT1 is a member of the NAD+-dependent histone deacetylase family and participates in several molecular processes, including mitochondrial biogenesis, endoplasmic reticulum stress, and the intrinsic apoptotic pathway [5]. SIRT1 plays a role in HIV infection by deacetylating HIV-1 Tat at the lys50 residue, facilitating the transactivation of the viral genome within the host cell [37]. In fact, SIRT2 and SIRT3 also have the capacity to deacetylase this viral protein [5,37] due to their conserved catalytic domain [30]. Likewise, HIV-1 Tat can also inhibit SIRT2 and SIRT3 [30,37]. The function of these sirtuins is related to their subcellular localization since SIRT1 predominantly localizes in the nucleus, whereas SIRT2 resides in the cytoplasm, and SIRT3 is one of the mitochondrial sirtuins [5].

While it is well documented that glial cells play a significant role in neurodegeneration due to their involvement in defective cell maintenance and the release of neurotoxic molecules, our understanding of the specific molecular pathways impacted within neurons by HIV infection remains incomplete. In this study, we aimed to investigate alterations in gene expression and telomerase activity related to mitophagy and mitochondrial biogenesis in hippocampal neurons exposed to the recombinant HIV-1 Tat protein. To achieve this, we utilized an animal model that simulates the effects of HIV-1 Tat protein exposure in the brain. By exploring these changes at the genetic level, we expect to shed light on the intricate mechanisms underlying neurodegeneration in the context of HIV infection.

## 2. Results

### 2.1. Expression of Markers of Mitochondrial Dysfunction in Hippocampal Neurons

**Figure 1:**
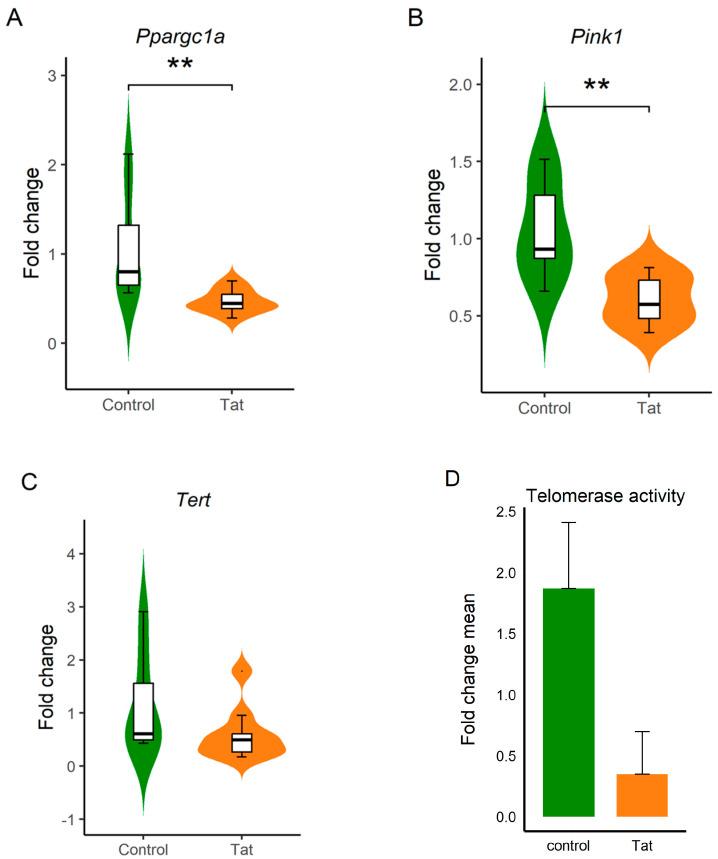
Expression of markers of mitochondrial dysfunction in the hippocampal neurons of Sprague–Dawley rats exposed to recombinant HIV-1 Tat (Tat group) and the control group (control). (**A**) Expression of *Ppargc1a*. (**B**) Expression of *Pink1*. (**C**) Expression of *Tert*. Data are presented as 2^−∆∆Ct^ distribution as a violin and boxplot showing the range 25th to 75th percentile and the median (Control group, *n* = 7; Tat group, *n* = 9). (**D**) Telomerase activity. Data are presented as the mean ± SEM. (Control group, *n* = 5; Tat group, *n* =5). ** p < 0.01.

To analyze whether HIV-1 Tat induces alterations in mitochondrial biogenesis and mitophagy, we assessed the quantification of the gene expression of *Ppargc1a* and *Pink1*. The neurons from the hippocampus exposed to the viral protein showed statistically significant downregulation of *Ppargc1a* and *Pink1* (*p* < 0.01) compared to the controls (Figure 1). *Ppargc1a* and *Pink1* mRNA were reduced by 2.26 and 1.73-fold, respectively, due to the presence of HIV-1 Tat.

We evaluated telomerase expression and activity. Interestingly, our results indicate that the gene expression of *Tert* was not significantly different between the experimental and control groups. Meanwhile, the activity was undetectable in neuronal cells in subjects of the experimental group compared to the control group (Figure 1).

### 2.2. Expression of Sirtuins in Hippocampal Neurons

**Figure 2:**
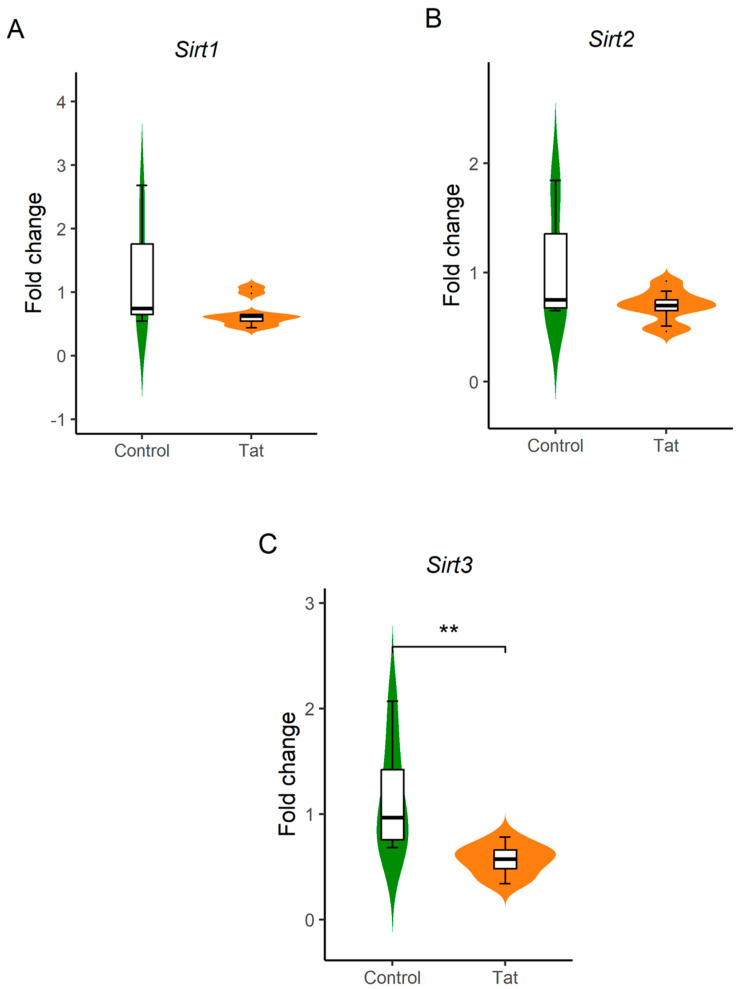
Expression of sirtuins 1-3 in the hippocampal neurons of Sprague–Dawley rats exposed to recombinant HIV-1 Tat (Tat group) and the control group (control). (**A**) Expression of *Sirt1*. (**B**) Expression of *Sirt2*. (**C**) Expression of *Sirt3*. Data are presented as 2^−∆∆Ct^ distribution as a violin and boxplot showing the range 25th to 75th percentile and the median (Control group, *n* = 7; Tat group, *n* = 9). ** p < 0.01.

**Figure 3:**
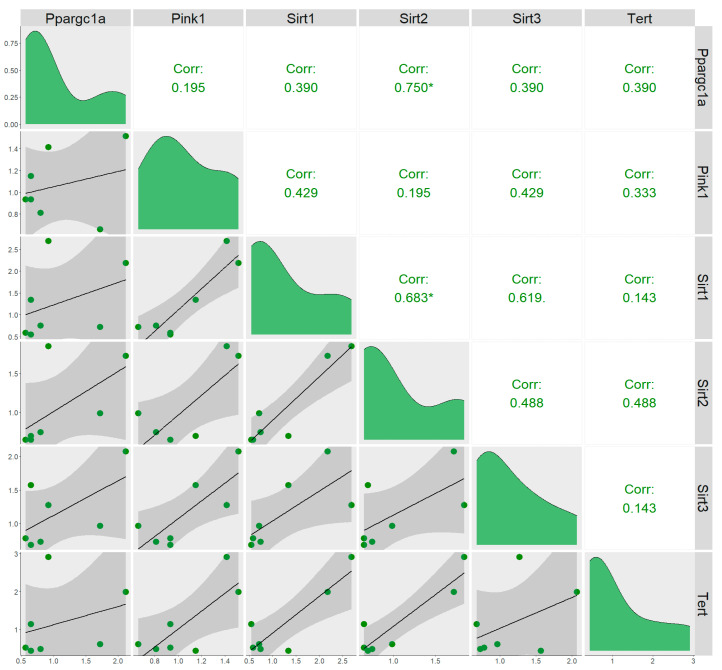
Kendall’s correlation between mRNA levels of the control group. The distribution of the data is exhibited in the diagonal. Scatter plots are displayed in the lower position of the diagonal. In the upper position, the correlation, tau-value, for both groups is indicated. The *p*-values are presented as * *p* < 0.05.

**Figure 4:**
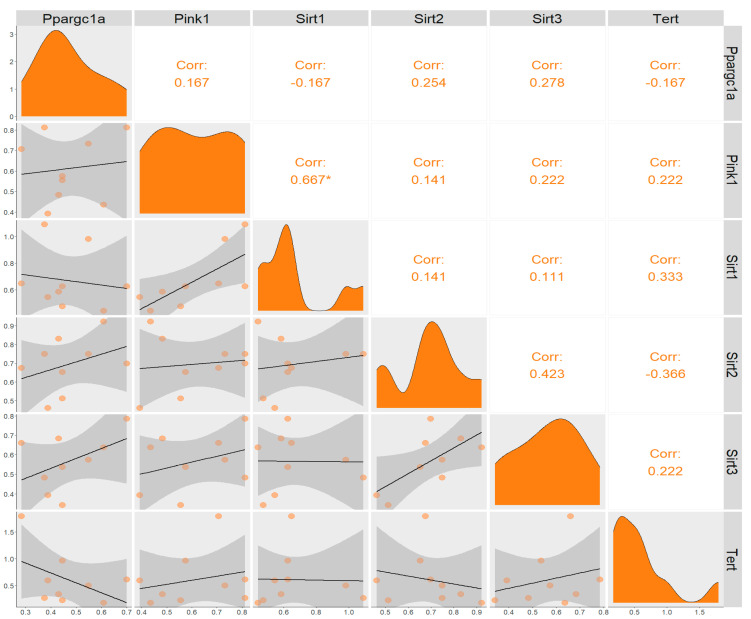
Kendall’s correlation between mRNA levels of the experimental group. The distribution of the data is exhibited in the diagonal. Scatter plots are displayed in the lower position of the diagonal. In the upper position, the correlation, tau-value, for both groups is indicated. The *p*-values are presented as * *p* < 0.05.

We subsequently determined the expression of *Sirt1, Sirt2* and *Sirt3*. Notably, only the mitochondrial sirtuin 3 was significantly reduced by 2.04-fold in the HIV-1 Tat-exposed group (*p* < 0.01) in comparison with the control (Figure 2). Additionally, we found that both *Sirt1* and *Sirt2* gene expressions were strongly positively correlated with the controls (tau = 0.683) (Figure 3). At the same time, Sirt2 is strongly and positively correlated with *Ppargc1a* in the same group (tau = 0.750) (Figure 3). Meanwhile, *Sirt1* and *Pink1* expression exhibited a strong positive correlation within the experimental group (tau = 0.667) (Figure 4).

## 3. Discussion

**Figure 5:**
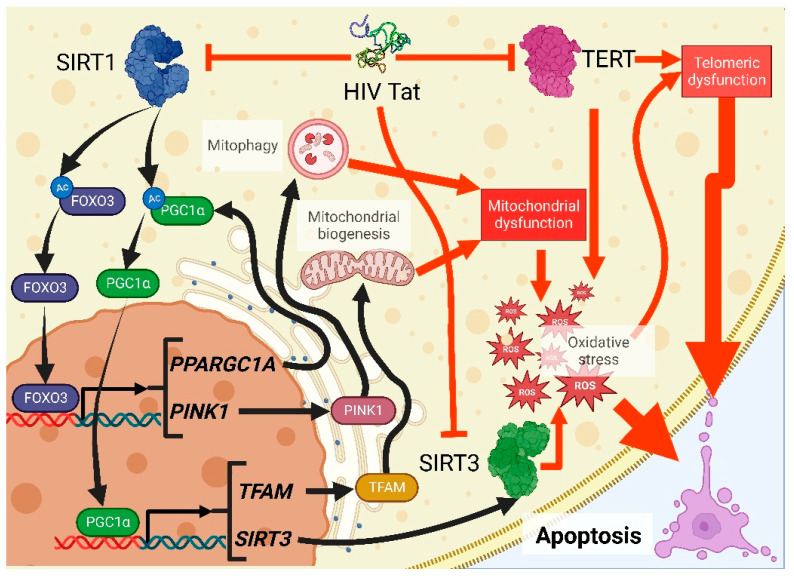
Mechanisms of HIV-Tat-induced mitochondrial dysfunction in the neuron. HIV-Tat inhibits SIRT1, avoiding the deacetylation of FOXO3. Acetylated FOXO3 cannot bind to the promoter regions of genes *Pink1* and *Ppargc1a*, reducing their transcription and mRNA levels. SIRT1 deacetylase PGC1a, allowing this protein to promote the transcription of TFAM and SIRT3. HIV-Tat-induced inhibition of SIRT1 impacts the mRNA levels of SIRT3 via PCG1a and FOXO3. Mitophagy and mitochondrial biogenesis are compromised, leading to an increase in ROS. HIV-Tat inactivates SIRT3, affecting the mitigation of ROS. The elevated ROS levels cause oxidative stress, contributing to telomeric dysfunction. HIV-Tat reduces the telomerase activity, obstructing its participation in the ROS amelioration. Mitochondrial dysfunction, oxidative stress, and telomeric dysfunction lead to apoptosis.

Mitochondrial dysfunction is a crucial feature in the landscape of neurodegenerative diseases [22], including HAND [23]. Hence, our investigation delved into markers associated with mitophagy and mitochondrial biogenesis, specifically PINK1 and PGC1a, respectively. The expression of PINK1, an elemental kinase for mitophagosome formation and the subsequent elimination of damaged mitochondria [38], exhibits an increase in neurons upon exposure to HIV-1 Tat [26]. Likewise, in mouse microglia, this viral protein induces PINK1 expression 24 h after exposure, suggesting mitophagy initiation. Nonetheless, at the 48 h mark, microglia exhibited a decrease in the expression of this gene [18], aligning with our in vivo observations in hippocampal neurons exposed to recombinant HIV-1 Tat. The observed time-dependent variation may be related to the Forkhead box protein O3 (FOXO3), a transcription factor for PINK1, whose activation depends on SIRT1 [39]. Hence, given the fact that HIV-1 Tat inhibits this sirtuin in a time-dependent manner [30,40], it is plausible to attribute the reduction in *Pink1* to the inactivation of FOXO3 via SIRT1 (Figure 5). Therefore, compounds such as SRT1720, which act as activators of SIRT1, have the potential to increase the mRNA and protein levels of PINK1 in macrophages [39]. As a result, it is reasonable to infer that HIV-1 Tat compromises the SIRT1-dependent deacetylation of FOXO3, leading to decreased transcription of genes, including *Pink1* (Figure 5).

Furthermore, it is imperative to consider that HIV-1 Tat-induced inhibition of SIRT1 does not occur at the onset of infection. Instead, it is associated with the intracellular concentration of the viral protein [37], which in turn is influenced by the time-dependent internalization of this protein [15]. It has been demonstrated that deacetylation of the PINK1/Parkin complex by SIRT3 is necessary to initiate mitophagosome formation. HIV-1 Tat not only inhibits SIRT1, as mentioned previously, but also hinders the deacetylation function of SIRT3 [30]. Consequently, this leads to a reduction in *Pink1* expression, as observed in this study, and an increase in the acetylation of the protein. Furthermore, high acetylation levels are associated with the activation of the intrinsic apoptosis pathway [41]. Thus, the decrease in *Pink1* leads to the inability of neurons to create mitophagosomes and thereupon, to the accumulation of damaged mitochondria in hippocampal neurons.

On the other hand, to counteract mitochondrial dysfunction, cells can employ two strategies: the elimination of damaged mitochondria, as mentioned above through mitophagy, and the generation of a novel organelle, known as mitochondrial biogenesis [34]. In reference to HIV, PGC1a, an essential transcription factor for the generation of new mitochondria, has been observed to be reduced in the frontal cortex of individuals diagnosed with HAND [36]. Our results revealed that *Ppargc1a* expression is reduced in the presence of HIV-1 Tat in rat hippocampal neurons. In this way, neurons exposed to this viral protein are unable to generate more mitochondria, so cell energy metabolism is compromised, as observed by Darbinian et al., where ATP levels were found to be significantly reduced in exposed neurons, which leads cells to activate the apoptosis pathway [26], thus contributing to neuronal loss.

The suppression of *Ppargc1a* transcription appears to be regulated by SIRT3, as Torrens-Mass et al. have shown that the absence of SIRT3 leads to a decrease in PGC1a expression [35]. Our results revealed that levels of *Sirt3* mRNA were also reduced in the hippocampal neurons of the experimental group compared to the control, which is consistent in mouse microglia, where HIV-1 Tat decreases both the enzymatic activity of SIRT3 and the levels of its mRNA and protein [27]. SIRT3 regulates *Ppargc1a* by deacetylating mitochondrial FOXO3 [35,42,43]. Thus, the inhibition of sirtuins 1 and 3 by HIV-1 Tat prevents PGC1a from binding to the promoter region of mitogenesis-related genes (Figure 5).

The inefficient mitochondrial quality control process, coupled with the inability to generate new organelles, drives the accumulation of high concentrations of ROS within the cell [38]. This condition causes the translocation of TERT to the mitochondria, where apart from its canonical role in maintaining telomere length, it also participates in apoptosis, mtDNA repair, and mitigating oxidative stress [44]. Therefore, we investigated the relationship of TERT with HIV-induced mitochondrial dysfunction. We found that in vivo *Tert* mRNA levels remain unaffected by HIV-1 Tat, contrary to the findings reported by Hsiao et al. in peripheral blood mononuclear cells of individuals with HIV. Interestingly, this research team reported no changes in TERT expression in microglia exposed to HIV-1 Tat [45], aligning with our results in neurons. This suggests that the impact of HIV on telomerase expression may vary depending on cell type and other viral factors, since TERT expression levels and regulatory mechanisms in the CNS differ from those in other tissues [46].

As no change in expression was observed in rat hippocampal neurons, the activity of TERT was determined. The results revealed that exposure of neuronal cells to HIV-1 Tat decreases telomerase activity, as demonstrated by Thangaraj et al. in mouse microglia [27]. The HIV-1 Tat-induced inactivation of TERT could be mediated in part by the inhibition of SIRT1 in the same context because this deacetylase is involved in the maturation of the TERT protein by deacetylating poly(A)-specific ribonuclease (PARN). This exonuclease plays a primary role in modifications of TERC, which is part of the telomerase complex [47]. TERT in neurons maintains a non-canonical role dependent on the balance between oxidant and antioxidant molecules [48,49], meaning that the reduction in telomerase activity in the CNS could be associated with the inability to mitigate mitochondrial ROS. In vitro studies are currently underway using compounds that enhance mRNA and protein levels, as well as the activity of this enzyme to protect neurons under conditions of Alzheimer’s disease [48], so in the field of HAND, it is feasible to carry out new research aimed at testing these drugs.

Under these circumstances of mitochondrial damage and increased oxidative stress, telomeric dysfunction develops, which contributes to the downregulation of PINK1 and the disruption of mitophagy. Additionally, telomeric dysfunction triggers the activation of p53 expression, which binds to the PGC1a promoter, thereby reducing its mRNA and impacting mtDNA transcription and replication [50]. In turn, SIRT3 expression mediated via PGC1a [51,52,53] might be compromised and consequently, both expressions decrease. Considering that SIRT3 regulates genes controlling the antioxidant response [54,55], a decrease in the expression levels of this sirtuin results in an upsurge of cellular ROS levels, activating the intrinsic apoptosis pathway [56] (Figure 5), and ultimately leading to neurodegeneration.

## 4. Materials and Methods

### 4.1. Animals

Male Sprague–Dawley rats were obtained from the Centro de Investigacion Biomedica de Occidente, del Instituto Mexicano del Seguro Social (IMSS). A total of 26 animals were included (408 ± 12.3 g average weight and 16.6 ± 1.3 average weeks old). There were 12 and 14 animals within the control and experimental group, respectively. All rats were housed in standard cages and had free access to commercial food and water. The conditions in the animal housing facility were 12 h light/dark cycles and a room temperature of 22–25 °C. During the stereotaxic surgery, animals were anesthetized with 72 mg/kg ketamine and 10 mg/kg xylazine. After 48 h of the stereotaxic intervention, all animal euthanasia was performed under anesthesia, and the animals were promptly decapitated.

### 4.2. Tat Injection

The cranial surgeries were performed in a digital rat stereotaxic instrument (Stoelting Co., Wood Dale, IL, USA). All animals were kept at 37 °C by using a heat pad during the procedure. The experimental group was injected with 100 ng of recombinant HIV-1 Tat clade B protein (Prospec-Tany Technogene Ltd. #HIV-129, Ness-Ziona, Israel) in 1 µL of saline, and for the control group, 1 µL of saline was used. The bilateral hippocampus injections were performed with a 2 µL microsyringe with 30 gauge (Hamilton Co., Reno, NV, USA). The coordinates to reach the dentate gyrus were −3.8 mm posterior to the bregma, ±1.8 mm left/right from the sagittal suture, and −3.7 mm from the surface of the skull according to the Paxinos and Watson rat brain atlas [57]. Following the surgical procedure, every animal was placed in a separate enclosure with ad libitum access to food and water.

### 4.3. Tissue Collection, Cell Suspension and Neuron Isolation

The rat hippocampus was collected and immediately processed for cell dissociation using a NeuroCult^TM^ Enzymatic Dissociation Kit (StemCell Technologies, Vancouver, BC Canada) following the instructions from the manufacturer.

Hippocampal rat neurons were isolated using positive immunomagnetic cell separation using rat microbeads CD90 (Miltenyi Biotec, Bergisch Gladbach, Germany) according to the MACS^®^ Technology protocol, where the cell suspension was incubated with the microbeads and then placed into MACS^®^ Columns and a strong permanent magnet MACS^®^ separator.

### 4.4. Relative Gene Expression

RNA extraction from the cell suspension was performed with a RNeasy Plus Mini Kit (Qiagen, Hilden, Germany) following the supplier’s instructions. The concentration and integrity of total RNA were measured by the wavelength of 260 nm and with the ratio of 260 nm/280 nm and 260 nm/230 nm in a Nanodrop One/One^C^ (Thermo Fisher Scientific, Waltham, MA, USA). Reverse transcriptase-quantitative polymerase chain reaction (RT-qPCR) was performed using QuantiTect SYBR Green RT-PCR Kit (Qiagen) and Rotor-Gene Q (Qiagen). The expression of each target gene was normalized to Gapdh. The primers used in the assays were QuantiTect Primer Assay of Qiagen (Rn_Pink1_1_SG QT01618379, Rn_Ppargc1a_1_SG QT00189196, Rn_Tert_1_SG QT00412846, Rn_Sirt1_3_SG QT01808345, Rn_Sirt2_1_SG QT01629138, Rn_Sirt3_3_SG QT01761585, and Rn_Gapdh_1_SG QT00199633. Data are presented as the fold change obtained by the 2^−∆∆Ct^ method.

### 4.5. Telomerase Activity

The telomerase activity was quantified according to the manufacturer’s instructions. A Telomerase Activity Quantification qPCR Assay Kit (ScienCell, Carlsbad, CA, USA) was used, and all assays were performed in Rotor-Gene Q (Qiagen). Protein was extracted from each sample using a lysis buffer with 0.1 M PMSF in isopropanol and β-mercaptoethanol. Telomerase reaction and qPCR were performed according to the kit protocol. Telomerase relative activity was measured employing the 2^−∆Ct^ method using the mean of control Ct. It was considered that Ct > 33 was an undetectable telomerase activity.

### 4.6. Statistical Analysis

All statistical analyses were performed using RStudio version 4.3.1 (PBC Boston MA). Normality was assessed using the Shapiro–Wilk test. Differences between the control and experimental groups were evaluated employing the Mann–Whitney U test, considering a significance value of *p* < 0.05. Kendall’s correlation was used to associate the gene expression within the groups; a strong correlation was considered with a tau-value > 0.60 and *p*-value < 0.05, tau = −1 was a negative correlation, and tau = 1 was a positive correlation. The sample size was not predetermined using any statistical method. Gene expression data are shown as medians and interquartile ranges (IQRs) and are illustrated in violin graphs.

## 5. Conclusions

HIV-1 Tat has effects on mitochondrial processes at the gene expression level in hippocampal neurons. It could disrupt mitophagy and mitochondrial biogenesis by reducing the expression of *Pink1* and *Ppargc1a*, respectively. This dysregulation is characterized by the downregulation of *Sirt3*. Furthermore, HIV-1 Tat could influence the modulation of ROS by decreasing the expression of *Sirt3* and reducing telomerase activity.

Despite the current findings revealing mitochondrial dysfunction linked to oxidative stress, the methodology used in this study requires further studies such as protein acetylation levels and organelle metabolism assays, to confirm the impact of HIV-1 Tat on mitochondrial homeostasis.

## Data Availability

All data for this study are presented within the article. Raw data are available from the corresponding author upon request.
